# Identification of Benzodiazepine Use Based on Dried Blood Stains Analysis

**DOI:** 10.3390/ph17060799

**Published:** 2024-06-18

**Authors:** Lucía Fernández-López, Sandra Rodríguez, Alberto Cánovas-Cabanes, Francisco-Javier Teruel-Fernández, Pilar Almela, Juan-Pedro Hernández del Rincón, Javier Navarro-Zaragoza, María Falcón

**Affiliations:** 1 Department of Pharmacology, Faculty of Medicine, University of Murcia, 30120 Murcia, Spain; lucia.fernandez2@um.es (L.F.-L.); palmela@um.es (P.A.); 2 IMIB-Arrixaca, Instituto Murciano de Investigación Biosanitaria, 30120 Murcia, Spainjphrincon@um.es (J.-P.H.d.R.); falcon@um.es (M.F.); 3 Forensic and Legal Medicine, Faculty of Medicine, University of Murcia, 30120 Murcia, Spain

**Keywords:** benzodiazepines, dried blood stain, overdose, toxicological effects, drugs

## Abstract

Biological matrices are typically used in forensic toxicological or pharmacological analysis: mainly blood, vitreous humor or urine. However, there are many cases in which crimes are a consequence of drug intoxication or drug abuse and they are not closed because over the months or years the samples become altered or decomposed. A dried blood stains test (DBS-MS) has recently been proposed to be used in drug toxicology when blood is found at a crime scene. This test could help an investigator to reveal what a person had consumed before the perpetration of the crime. In order to check the possibilities of this test, we analyzed several dried blood stains located on a cotton fabric. Therefore, the aim of this study was to determine if the analysis of a dried blood spot located on a cotton fabric could be an alternate source of obtaining toxicological results, particularly regarding benzodiazepines. We splashed blood stains on cotton fabric with different concentrations of the following benzodiazepines: alprazolam, bromazepam, clonazepam, diazepam and lorazepam, which were dried for 96 h and subsequently quantified by high-performance liquid chromatography coupled mass spectrometry (HPLC-MS). Our results show that it is possible to identify several benzodiazepines contained in a cotton fabric blood stain; consequently, this method may add another sample option to the toxicological analysis of biological vestiges found at a crime scene.

## 1. Introduction

It is known that being under the influence of alcohol, drugs and/or substances of abuse is common when a crime is committed. Moreover, when the concentrations of these substances are quantified at toxic levels, this fact is considered as extenuating circumstances, which result in a lower charge or a reduction in punishment. Under certain conditions, it is impossible to perform screening tests or to establish previous drug/alcohol use, especially when the criminal is not found. Similarly, if the crime was committed years ago new methods and/or matrices are clearly necessary. Alcohol, cocaine, opioids, marijuana or benzodiazepines are among the substances of abuse most commonly consumed by criminals [1]. Benzodiazepines are one of the most abused drugs worldwide, singularly in developed countries. Particularly, they have a wide use in the detoxification of several drugs of abuse. Both people who are involved in criminal facts who usually have been incarcerated before and people who have been included in detoxification of substance abuse programs, are often benzodiazepine users [2]. Besides, the administration of benzodiazepines as recreational drugs is associated with misuse and dependence. In fact, this substance is linked to the appearance of drug dependence at different ages, but also to a high number of overdose deaths usually caused by the concomitant use of other drugs or alcohol [3].

According to the statistics published by the World Health Organization (WHO), benzodiazepines are one of the three most widely administered drugs in several countries. It is important to highlight that although benzodiazepines should be prescribed by physicians, they are often acquired without a prescription [4]. Mostly, the countries with the highest consumption rates are within Europe and America [5].

Benzodiazepines are psychoactive substances used for the treatment of generalized anxiety disorder and insomnia [6]. They behave as modulators of the GABA receptor, which is located in the postsynaptic terminal, by enhancing the inhibition of the central nervous system and generating a selective depression [7]. Regarding the metabolism of benzodiazepines, they are distributed throughout all the tissues, crossing even the placental barrier and the blood-brain barrier. They are administered orally, intravenously or intramuscularly, and metabolized in the liver by microsomal enzymes. Finally, the excretion of benzodiazepines is controlled by the kidney [7]. The most prescribed benzodiazepines are diazepam, lorazepam, alprazolam, temazepam, chlordiazepoxide, nitrazepam, triazolam, flunitrazepam and lormetazepam [8]. One of the biggest issues related to benzodiazepines is that they generate a wide number of adverse drug events. These events are usually a result of an interaction between different drugs, i.e., with antidepressants, barbiturates or alcohol. Sometimes, adverse drug events can even lead to death as a consequence of a synergic mechanism. Peculiarly, this mixture produces an enhancement of the depressant effects on breathing and consciousness, even leading to death or a coma. Additionally, if the benzodiazepines are administered intravenously (IV) they can produce ataxia, drowsiness, nystagmus, dysarthria, myotic pupils or hypothermia [9]. Furthermore, benzodiazepines are the most detected drugs in toxic-chemical analysis related to chemical submission. They frequently appear in cases of alleged crimes against sexual freedom [10]. According to the official statistics published by the International Narcotics Control Board (INCB), a great number of registered overdose deaths were also due to benzodiazepines’ intervention or contribution [11]. Benzodiazepines are a key factor in pharmacology, toxicology and forensic analysis, since they are involved in multitude of crimes, adverse events and overdose situations [1,7]. Despite this, at this moment there are no methods available to quantify benzodiazepines in a crime scene if the body is absent. In contrast, it is very common to find other evidence and, very often, dried blood. Bloodstains can help with crime scene reconstruction or to identify people by DNA analysis [12,13]. We have adapted the term dried blood spot (DBS) to dried blood stain qualitative analysis by HPLC-MS (DBS-MS) to be correctly differenced from the clinical use. Forensic and pharmacological use could be important, because finding new matrices to identify benzodiazepines and, even in the future, to quantify concentration levels of benzodiazepines in order to diagnose intoxication or overdose would be essential. Nevertheless, qualitative analysis could help to reveal what a person had consumed before the crime had been committed, meaning these results could be used as extenuating circumstances in a trial [14,15].

This technique is based on the dried blood spot analysis (DBS) that has been used in previous studies for a wide range of applications in clinical practice, basic research and population-based research [16,17,18,19,20,21]. The use of DBS as a sampling technique represents an easy method which shows reliable quantitative results months after sample collection and the possibility to perform an analysis from a very small sample volume [21]. One of the most common uses of DBS is in regard to newborn screening programs for the detection of metabolic disorders [22]. On the other hand, previous research has focused also on HIV surveillance, therapeutic drug monitoring and clinical chemistry [23,24,25,26].

DBS analysis has several advantages: the sample collection is easy and simple, it is a minimally invasive technique which needs a small sample volume and the analytes are easy to transport and store. During this century, the technique has been adapted and new methodology has been validated. There is an exponential growth of publications on scientific applications in various fields of research [16]. In contrast, there is not much literature about the potential adaptation of DBS for drug use screening, which could help to elucidate if the criminal had consumed any drugs and its subsequent relevance to the case. The dried blood stain test has been widely performed in hospitals by using special cards, and only once before to test for benzodiazepines [27].

Since, to the best of our knowledge, there are only a few references in the literature on the use of a dried blood spot test for testing the blood found in cotton fabric or even on a different clothing fabric [27,28], we have adopted and widen this term (DBS-MS) for drug screening in cotton fabric instead of on cards. Our proposal allows the possibility of analyzing several compounds per single run. Consequently, the aim of this research was to analyze blood stains dried after 96 h (4 days) on cotton fabric to identify the following benzodiazepines: alprazolam, bromazepam, clonazepam, diazepam and lorazepam (the most prescribed benzodiazepines in Spain in the last years), by using high-resolution liquid chromatography coupled mass spectrometry (HPLC-MS) to obtain qualitative and quantitative values that could help for a pharmacological, medical and toxicological analysis.

## 2. Results

**Figure 1:**
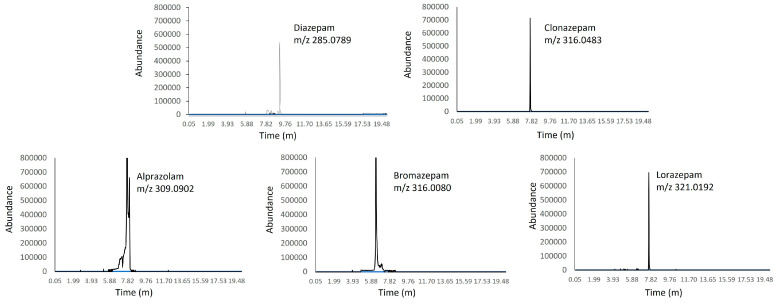
Representative chromatograms obtained following the extraction of blood stains spiked with alprazolam, bromazepam, clonazepam, diazepam and lorazepam at 10 µg/mL blood.

Our results showed that each benzodiazepine that we planned to analyze in this research was detected. Indeed, this fact demonstrates the qualitative value of this study. Representative chromatograms that were obtained after the extraction of the blood stains which were spiked with alprazolam, bromazepam, clonazepam, diazepam and lorazepam at 10 µg/mL blood are shown in Figure 1. The run was completed in 20 min. Additional peaks due to endogenous substances that could interfere with the detection of the compounds of interest were not observed. The small peaks that appeared in the graphs around the signal of the corresponding analytes were considered background noise or unimportant impurities due to their minimal abundance compared to the analytes. Furthermore, no trace of carryover was found in the blank samples injected after the highest point of the calibration curve. Thus, these results show that the benzodiazepines contained in dried blood stains found on a cotton fabric can be detected after 96 h, suggesting they are an alternative sample for toxicological analysis.

Regarding the quantitative value of the study, linear calibration curves for all the benzodiazepines analyzed by the dried blood stain test arose determination coefficients (R^2^) results over 0.99. The statistical significance of intercept in calibration curves was *t* = 2.54; *p* = 0.044 for alprazolam; *t* = 4.53, *p* = 0.02 for clonazepam; *t* = 7.94, *p* = 0.004 for diazepam; *t* = 2.88, *p* = 0.044 for bromazepam; and *t* = 5.89, *p* = 0.001 for lorazepam. Besides, the LOD and LOQ of each analyte were calculated (Table 1). The limit of quantification (LOQ) objective was selected as the lowest concentration level for which the method was fully validated by using spiked samples with satisfactory recovery (between 70 and 120%) and precision (relative standard deviation (RSD) ≤ 20%). The limit of detection (LOD) was defined as the lowest concentration that the analytical process could reliably differentiate from background levels. It was estimated for a signal-to-noise of three from the quantitation chromatograms of samples were spiked at the lowest analyte concentration tested. The precision and accuracy values obtained from the QC samples are shown in Table 2. Unfortunately, the QCL samples began to form a clot and they had to be discarded from the study. According to the precision values, they were under 15% in QCM for clonazepam or lorazepam (and alprazolam 15.1%) as recommended in the validation guidelines, but clearly over 15% for bromazepam and diazepam. A QCH over 15% was only diazepam (and clonazepam, 15.8%). On the other hand, the accuracy values were better at medium concentrations for bromazepam, clonazepam and diazepam (<9%), while for alprazolam and lorazepam the values were more acceptable at higher concentrations (<11%).

## 3. Discussion

The method proposed in this research has both advantages and limitations; nevertheless, the importance of the project lies in the novelty of the subject matter. Our concern is that there is no published literature regarding the analysis of blood stains on cotton fabric. Previous research related to DBS analysis is focused on the identification of drugs using cards for blood spots [22,25,29,30,31]. However, in pharmacology and toxicology, the study of DBS-MS on cotton fabric is of greater interest and more useful than by cards. The reason is that in a crime scene, blood stains are usually found on clothes, towels, blankets, tablecloths, curtains, etc. If this technique is performed, it will be not mandatory to find the corpse or the criminal in order to know whether benzodiazepines have been consumed by or administered to someone.

As blood has been used very often for the monitorization of drug use and abuse over the last decades, its importance in toxicology has been widely demonstrated. The presence of blood at a crime scene also provides valuable information that can be decisive in solving a case. Nowadays, a luminol test is widely used in criminal investigation in order to detect traces of blood, i.e., at a crime scene or after a suicide. The blue colored chemiluminescence of luminol is catalyzed by hemoglobin in the blood. The luminol reagent (5-amino-2,3-dihydro-1,4-phthalazinedione) is responsible of the reaction. This reaction is widely used in research to find bloodstains. The emission of light is almost instantaneous when the luminol gets in touch with the blood. Luminol can be used to detect blood even after washing or dilution. It is possible to detect bloodstains of more than six years old on washed surfaces, in large rooms and on different supports. Indeed, a luminol test can detect blood in a cotton fabric and is compatible with serological and DNA determination, making possible the performance of the dried blood stain method [12].

However, DBS-MS offers several advantages compared with other blood collection techniques. First of all, this method is quite simple, quick and, of course, is a minimally invasive technique that minimizes the risk of infection associated with the handling of the samples. Besides, it is a dry matrix and therefore the absence of fluids promotes the inactivation of pathogens, including HIV and hepatitis virus [31,32]. Furthermore, this method allows us to know in real time the amount of a particular drug in the blood, reducing the error that occurs, for example, with hair samples. The DBS-MS test is also useful in cases where the victim reports the event a few days later, for example, a robbery or rape related to chemical submission. It is known that there are a lot of cases where the victim is confused at the beginning but finally decides to report to the police. The problem is that delayed reporting facilitates the drug being metabolized and excreted [33]. Another advantage of the DBS-MS test would be related to traffic matters, i.e., cases of driving under the influence of drugs, or after crashes. Nevertheless, only authorized medical are allowed to carry out this sample collection; therefore, police and the subjects must wait until the authorized personnel arrive. This period of extra time can often lead to the metabolization of the drug; consequently, the results obtained would not reflect the blood concentration in real time [34].

We would also like to mention that unlike fresh blood collection, which requires the detection of substances of abuse to be carried out shortly after the crime in order to avoid the drug metabolism [13], drug concentrations in DBS-MS are not affected by natural processes of metabolism and elimination (in case of alive people), or degradation and putrefaction (in case of dead people). Dried blood stains and their content can remain stable for longer on cotton. The increased stability of many analytes allows the investigation to be carried out a long time after the exposition, leaving the time factor out of the scene [12]. This is particularly interesting in the case of benzodiazepines since different reports have revealed their low stability under different storage conditions. The main reason for this instability is the presence of an amide group in their structure which facilitates hydrolysis reactions. In contrast, the dried blood stain test is performed in a dry matrix which reduces both microbial degradation and enzymatic activity [21,35,36,37].

Moreover, it is worth noting the fact that it would not be necessary to find the individual or the corpse in order to carry out a drug test. It could be possible to determine the substances that a person had consumed before the incident only by finding a blood stain on a textile surface at the crime scene. If blood belongs to the criminal, it would be possible to link a suspect to the scene of crime and to determine if he or she acted under the influence of drugs, if the victim was administered drugs or even if he or she died by an adverse drug event [8,9,12,14].

There are situations in which the detection of any substances would add value to the investigation, regardless of the concentration. Qualitative values are enough in a variety of cases: the detection of a poison, where the purpose is inherent; in case of the detection of a substance to which the victim was not aware of having been exposed; or in the control of an abstinence state and/or replacement medication intake in subjects with drug dependence problems. In this case, the collection of DBS-MS samples is not hampered by privacy invasion issues related to gender difference, which can often mean an unsupervised sample collection, increasing the possibilities of sample adulteration [16].

However, quantitative values become important for substances whose concentration determines the toxicity. In this study, quantitative analysis was also performed. Linearity was good (R^2^ > 0.99), RSD values were valid for validation of dried blood stain analysis concerning clonazepam and lorazepam (and probably diazepam). Bromazepam, clonazepam and diazepam had the better accuracy values at medium concentrations (<9%), while alprazolam’s and lorazepam’s high concentration values were more acceptable (<11%). These results are the first observed regarding the detection of drugs by the dried blood stain test, and the method might be improved to be also considered a quantitative method.

Finally, as said before, this field has many possibilities and further research is necessary, i.e., DBS-MS can help the field of pharmacology and toxicology by providing an alternative way to screen for toxics in the body. Methods that are used today for drug screening are effective, but the body can metabolize the drugs or convert them to different metabolites by natural processes what would not happen in case of DBS-MS [13,14,38]. However, the development of a new technique is difficult and there is a lot of research ahead. We might keep performing this methodology by testing other drugs of interest and the influence of different factors, such as the stability after a period of time longer than six months, or even the environmental circumstances.

## 4. Materials and Methods

### 4.1. Reagents

Methanol (methyl alcohol, anhydrous, ChromAR^®^ HPLC) was acquired from Macron Fine Chemicals™ (Waltham, MA, USA), and acetonitrile HPLC from Lab-Scan analytical sciences (Gliwice, Poland). The internal standards (IS) used were alprazolam, bromazepam, clonazepam, lorazepam, diazepam and 7-aminoclonazepam-d_4_ at 1.0 mg/mL in acetonitrile, purchased from Salars (Como, Italy) and Cerilliant trading house (Round Rock, TX, USA). The nylon syringe filters (RephiQuik Syringe Filter Nylon 13 mm–0.45 μm) were supplied by Labolan S.L. Material (Esparza, Spain). 

### 4.2. Blood Samples

Human blood specimens were supplied by the Regional Blood Donation Center (Murcia, Spain), certified with the recognition of negative results for the presence of benzodiazepines and drugs of abuse (stored at 5 °C). 

### 4.3. Preparation of Stock Standard Solution

The stock standard solution (SSS) (5 µg/mL) contained all the benzodiazepines that were quantified. It was prepared by pouring 5 mL of each 100 µg/mL solution and methanol until a final volume of 100 mL. In order to achieve a complete homogenization, heat and agitation were provided. From this SSS, we obtained two working solutions (WS) at 100 ng/mL and 10 ng/mL, both in methanol.

### 4.4. Preparation of Calibration Standards (CS) and Quality Controls (QC)

The calibration standards (CS) were prepared at 1, 10, 100, 1000 and 10,000 ng/mL blood by spiking drug-free blood pool with suitable amounts of the methanol solutions (Table 3). Previously, we performed a calibration curve with the internal standards. These concentrations covered both the therapeutic and lethal ranges of the different benzodiazepines. Three quality control (QC) samples were prepared in drug-free blood at 2500 ng/mL (high control, QCH), 250 ng/mL (medium control, QCM) and 5 ng/mL (low control, QCL) to check the method. All CSs and QCs presented the IS at a concentration of 2 μg/mL.

### 4.5. Blood Stains

**Figure 2:**
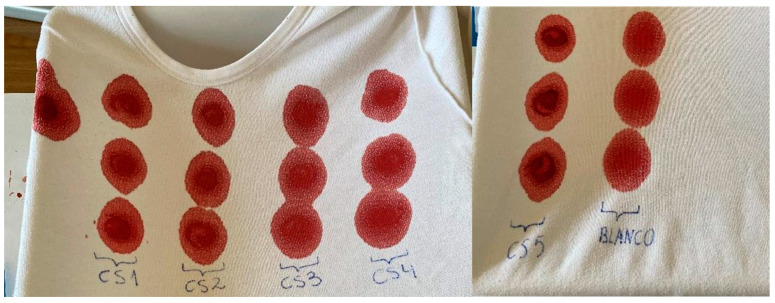
Five calibration standards (CS) and blank blood triplicated stains.

**Figure 3:**
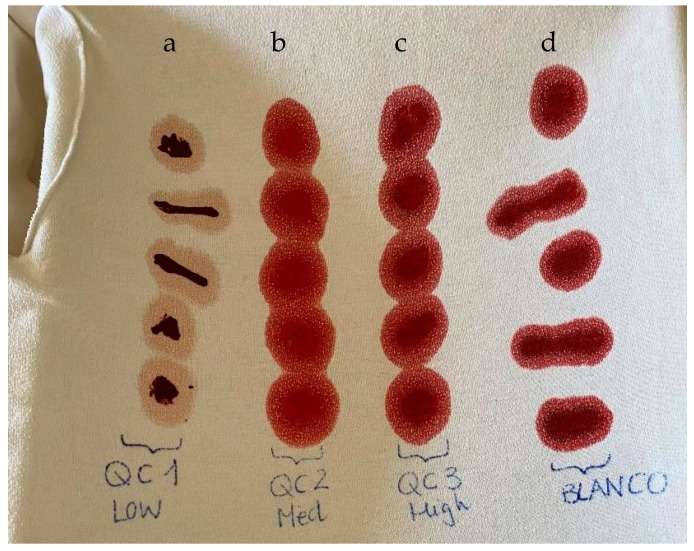
Quality control (QC) and blank blood quintupled stains. Column a: stains for QC1; column b: stains for QC2; column c: stains for QC3; column d: stains for blank analysis.

The blood stains were prepared by spiking 0.5 mL of each sample on a 100% cotton t-shirt. This method, as above is mentioned, is based on dried blood spots test. Stains for calibration procedures were made in triplicate and for quality controls in quintuplicate (Figure 2 and Figure 3). The stains were allowed to dry for 96 h at room temperature (±20 °C).

### 4.6. Extraction Procedure

A previously published method [20] was adapted to our case. Each dried blood stain was fragmented into tiny pieces (<1 mm) and introduced into a plastic tube. For each sample, 6 mL of a methanol/acetonitrile extraction solution (1:1) were added and shaken for one min. Afterwards, the samples were incubated in an ultrasonic bath for 15 min and vortexed for two minutes. The supernatant was collected and filtered with nylon syringe filters. The samples were stored at 5 °C until the analysis was performed.

### 4.7. HPLC-MS Conditions

A previously published method [24] was used to determine the working conditions of the HPLC-MS. The samples were thermostabilized at 4 °C and suspended on 200 μL of 0.1% formic acid + 10% acetonitrile. 20 μL per sample were injected into an Agilent (Santa Clara, CA, USA) HPLC-MS device (1290 Infinity series II HPLC coupled to a Q-TOF 6550 i-funnel mass spectrometer equipped with a Jet Stream dual ESI). The column used was a Zorbax Eclipse Plus C18 HPLC column (2.1 × 100 mm, 1.8 um) with a flow rate of 0.4 mL/min and a temperature of 40 °C. Solvents A (MiliQ water with 0.1% formic acid) and B (acetonitrile) were used for the separation of the compounds. After injection, the compounds were eluded with a gradient program starting from a 5% solution [B] which held for 15 min, followed by a 90% solution [B] which held for 2 min and returned to the initial condition of 5% [B]. The MS worked in positive mode. The pressure of the nebulizer gas was set at 40 psi, while the flow of the drying gas was set at 16 L/min at a temperature of 130 °C and the sheath gas was set at 11 L/min at 300 °C. The capillary spray, fragmentator, skimmer and octopole 1 RF Vpp were set at 3500 V, 100 V, 360 V and 750 V respectively. The mass spectra of the substances were recorded in the scan range 50–500 *m*/*z* to determine retention times and characteristic mass fragments of the compounds. Data analysis was performed with the MassHunter Qualitative Analysis Navigator software (Agilent Technologies, Rev.B.08.00). The qualifying ions monitoring and their retention time appear in Table 4. Extracted ion chromatograms corresponding to the transitions of the compounds listed in Table 4 were obtained and analyzed.

### 4.8. Validation Procedure

The method was subjected to a validation protocol following the most recently reported international criteria in forensic toxicology [39,40]. Selectivity, carryovers, linearity, limits of detection (LOD) and quantification (LOQ), precision and accuracy were determined in accordance with the criteria described in previous works [41,42,43,44]. The validation parameters were calculated using five different replicates of the three QC samples. Possible interferences due to endogenous compounds and among the substances aimed to study were analyzed. The evaluation of the interferences by endogenous substances depending on the texture was assessed by extracting drug-free blood stains and measuring the different peaks drawn in the chromatogram at the different retention times of the analytes and the IS. An aliquot of the extracted drug-free blood stains with only the IS added was injected immediately after the analysis of the highest concentration point of the calibration curve. This was made to discard carryovers at the retention times of the substances under investigation. Calibration curves were performed in triplicate for all the compounds, but the first set was discarded after coagulation during the process. To determine the LOD and LOQ for this method, five replicates of the blank samples were analyzed. The next step was to estimate the standard deviation (S.D.) for the mean noise level at the retention time window of each analyte to be used for the calculation (LOD = 3 S.D., LOQ = 10 S.D.). The precision and accuracy were analyzed at the three QC concentrations and expressed as standard deviation and error (%) of the measured values, respectively.

## 5. Conclusions

A new method for alprazolam, bromazepam, clonazepam, diazepam and lorazepam identification through dried blood stains located on cotton fabric after 96 h has been validated. The proposed method is simple, fast and effective. Drug identification in DBS-MS is not affected by natural processes of metabolism and elimination in vivo or degradation and putrefaction in case of death. In addition, this dry matrix ensures that enzymatic activity and microbial degradation are reduced. Therefore, the drugs remain stable longer than in the case of fresh blood, expanding the time frame for the investigation. Further research is necessary to improve the method and to adequate it to quantify the concentration of the benzodiazepines. In conclusion, we propose this method to be considered for the identification of benzodiazepines as an additional procedure to the usual scientific investigation.

## Figures and Tables

**Table 1:** Linearity results, LOD and LOQ values for analytes under investigation. LOD, limits of detection; LOQ, limits of quantification.

Analyte	Calibration Parameters			
	Equation	Determination Coefficients (r^2^)	LOD (ng/mL)	LOQ (ng/mL)
Alprazolam	y = 1850.3x + 663,736	0.991	0.5	1.5
Bromazepam	y = 739.49x + 103,379	0.999	0.5	1.7
Clonazepam	y = 145.17x + 51,295	0.997	0.2	0.6
Diazepam	y = 357.78x + 163,114	0.996	0.3	1.0
Lorazepam	y = 162.86x + 89,121	0.994	0.5	1.5

**Table 2:** Precision and accuracy values obtained for analytes under investigation. QCM, quality control medium; QCH, quality control high.

Analyte	RSD (%)		Accuracy (%)	
	QCM	QCH	QCM	QCH
Alprazolam	15.1	1.4	85.1	89.0
Bromazepam	23.7	4.5	88.8	93.0
Clonazepam	7.9	15.8	89.3	94.9
Diazepam	34.7	45.3	92.0	88.1
Lorazepam	1.1	13.4	107.3	109.5

**Table 3:** Calibration standards used for HPLC.

Calibration Standard (CS)	Concentration (ng/mL)	SSS, WS1, WS2 Volume (mL)	Blood Volume (mL)
CS1	1	0.5 WS2	4.5
CS2	10	0.5 WS1	4.5
CS3	100	0.1 SSS	4.9
CS4	1000	1 SSS	4
CS5	5000	0.25 of each 100 µg/mL solution	3.75

**Table 4:** Retention times (min) and characteristic ions (*m*/*z*) of analyzed substances by HPCL-MS.

Benzodiazepines	Precursor Ion [M + H]^+^ (*m/z*)	MS/MS (*m/z*)	Retention Time (min)
Alprazolam	309.0902	281.0721	7.72
Bromazepam	316.0080	182.0838	6.36
Clonazepam	316.0483	270.0554	7.67
Diazepam	285.0789	154.0418	9.12
Lorazepam	321.0192	275.0133	7.60
7-aminoclonazepam-d4	290.1055	274.0805	2.75

## Data Availability

Data available on request due to restrictions.
